# Participative leadership effects on followers’ radical creativity: the role of psychological safety and collaborative relationship

**DOI:** 10.1186/s40359-025-02950-3

**Published:** 2025-06-04

**Authors:** Guo Song, Md. Nurun Nabi, Md. Abu Issa Gazi, Mohammad Bin Amin, Abdul Rahman Bin S. Senathirajah, Md. Atikur Rahaman, Zhang Min

**Affiliations:** 1 School of Management, Jiujiang University, Jiujiang, 332005 China; 2 Department of Textile Engineering Management, Bangladesh University of Textiles (BUTex), Dhaka, 1208 Bangladesh; 3 Faculty of Business and Communications, INTI International University, Persiaran Perdana BBN Putra Nilai, Negeri Sembilan, Nilai, Negeri Sembilan, 71800 Malaysia; 4 Department of Business Administration, Faculty of Business Studies, Bangladesh Army University of Science and Technology, Saidpur, Nilphamari, 5310 Bangladesh; 5 School of Economics, Jiujiang University, Jiujiang, 332005 China; 6 Department of Business Studies, State University of Bangladesh, Dhaka, Bangladesh

**Keywords:** Participative leadership, Follower’s psychological safety, Collaborative relationship, Followers’ radical creativity, Social information processing theory, sustainable growth

## Abstract

This study indicates that there is still a limited number of studies that examined the nexus among participative leadership (PL), follower’s psychological safety (FPS), followers’ radical creativity (FRC), and collaborative relationship (CR) in the manufacturing sector of emerging economies. This paper emphasizes the importance of FPS and business collaboration to promote FRC and sheds new light on manufacturing stakeholders’ involvement. That is why this research aims to investigate the relationship between PL and FRC, including the mediating effect of FPS. Additionally, CR functioned as a moderator in the connection between FPS and FRC. This study applies the social information processing (SIP) theory as the theoretical background by bringing the dynamics between leaders and followers to the current scholarly discourse. A quantitative survey was conducted through a structured questionnaire from the samples, including 82 leaders and 240 followers from 178 manufacturing companies from all parts of Bangladesh. The researchers in this study employed SPSS for the descriptive statistics and PLS-SEM to evaluate the anticipated connections. Moreover, the quantitative results showed that PL has a positive and substantial relationship with both FPS and FRC (β = 0.25, t-statistics = 5.67, *P* < 0.001). Additionally, FPS has a mediation effect on the link between PL and FRC (β=-0.12, t-statistics = 22.59, *P* < 0.001) On the other hand, it has been found that the collaborative relationship’s moderating influence is insignificant (β = 0.29, T-values = 0.66, SE = 0.0000031). This study clarifies how PL and FPS develop the FRC in Bangladeshi industrial firms. This study contributes to the literature by highlighting how organizations can promote FRC among their followers, foster PS through CR, and motivate individuals to actively participate in FRC endeavors to boost organizational productivity and effectiveness.

**Supplementary Information:**

The online version contains supplementary material available at 10.1186/s40359-025-02950-3.

## Introduction

Creativity and innovation are essential sources of competitive advantage in today’s fast-paced workplace [1]. Organizations must defeat creative rivals and encourage innovation to survive and thrive in a world of ever-advancing technology, competitive pressures, and an unpredictable and highly volatile economic environment [2]. Organizations possess a strong understanding that their competitive success is contingent upon the ability of people or teams to generate innovative and useful ideas via collaborative efforts. Hence, it is essential to cultivate followers’ radical creativity (FRC) and promote entrepreneurial innovation to facilitate this transition [1]. In recent decades, there has been a significant advancement in the research field of creativity [3]. Besides, creativity encompasses the use of individuals’ talents, knowledge, skills, and experience to generate novel ideas for problem-solving, decision-making, and efficient task execution [4]. Pertinently, Zhang et al. [5] defined radical creativity as the creation or invention of innovative valuable concepts of visible or invisible products, or procedures. The idea of FRC focuses on the promotion of divergence uniqueness and the exploration of concepts that deviate from conventional ways and alternative approaches [5].

The development of followers’ creativity is not an innate process but rather needs the guidance and support of leaders. On the other hand, leaders are critical in fostering and promoting the development and growth of creativity and innovation [6]. Accordingly, researchers focused on fostering FRC in participative leadership (PL). Thus, a key question is how participative leaders affect followers’ psychological safety (FPS) for the followers’ creative behaviors and help to enhance their FRC through the collaborative relationship between the followers and leaders. On the other hand, when PL engage followers in decision-making, they feel more psychologically safe and secure, making collaborative and cooperative interpersonal social linkages more important [7]. Consequently, followers are more willing to share knowledge, take risks, and engage in creative undertakings. Conversely, in the case of PL, leaders consult with followers while making organizational decisions, which may be especially favorable to followers’ creativity. Busse and Regenberg [8] found a positive association between PL and follower involvement; whereas Chan [9] revealed that PL has significant impact on employment satisfaction.

Moreover, the phenomenon of radical follower creativity may be distinguished by the cognitive aspects of psychological empowerment, safety, and behavioral participation, as identified by Fu et al. [7]. Besides, the concept of FPS entails the collective idea that engaging in hazardous behaviors does not lead to adverse outcomes, allowing individuals to demonstrate initiative or undertake risks without fear of harm to their image. Individuals are more inclined to exhibit trust and cooperation in engaging in creative endeavors and generating innovative concepts if they perceive a sense of safety. In line with scholarly discourse, participatory leaders are known to create inclusive organizational cultures that promote the values of respect, trust, confidence, and acceptance [10]. According to Nabi and Liu [1], followers generate novel ideas for all categories of visible and invisible products, procedures, or production processes because of the leader’s input. These ideas have the potential to be lucrative. A considerable body of data indicates that the phenomenon of transformational leadership also referred to as FPS, has a major impact on the creative abilities of subordinates. However, there are contrasting shreds of evidence in the literature regarding the role of FPS, such as Njoroge et al., [11] found a partially mediated effect of FPS [12], demonstrated positive mediation results, and [13] finding no positive/significant mediating results. As a result, the role of FPS requires more investigation and needs further research [7, 14].

Notably, a collaborative relationship means collaboration in the workplace is a group of individuals exchanging their ideas and abilities to achieve a shared objective. Working together, compared to individually, increases productivity and offers followers a feeling of purpose in the firm [7]. Accordingly, cognitive views of collaborative relationships and behavioural involvement in a psychologically secure environment influence followers’ creativity. Furthermore, the PL style significantly influences employee creativity and is suitable for recognizing new opportunities and building business skills. PL boosts followers’ confidence and trustworthiness through the leader-follower collaborative relationship. Lythreatis et al. [15] underlined the need to examine moderating factors to predict employee creativity more. In addition [16], et al. [16]. stressed the need to investigate other moderating factors to build significant correlations between the results of PL and FRC relationships.

Previously Chen [10] concentrated on examining the relationship between PL and the creative abilities of followers and found a variety of results related to the impact of PL on the creative abilities of followers. On the other hand, Wang & Rode [17] examined various determinants of PL those affects several other factors. Further research is required to explore various factors related to PL that intrinsically motivate employees to engage in FRC [7, 14]. However, the presence of managers might potentially have practical implications for resource conservation [7]. In the field of organizational research, Li et al. [18] and Newman et al. [19] focused on the concept of PL as a means to understand and analyze the motivational and psychological mechanisms that have a direct or indirect influence on FRC. The emphasis needs to be more extensive and allure further investigation since FRC is a springboard for radical innovation of the textile organization.

In supporting the aforementioned relationship, Social Information Processing (SIP) theory elucidates the mechanisms by which cognitive and behavioral responses are fostered by social interactions with important individuals, including leaders and peers [10]. According to Li et al. [18] followers tend to gather valuable information from the words and actions of their leaders in order to form their perceptions of the workplace and adapt their behaviour based on the situational appropriateness of certain actions. It can be attributed to the leaders’ heightened visibility and active engagement with followers, as noted by Jin and Qing [14]. A participatory leader’s collaborative and dyadic interactions create a psychologically secure atmosphere where creative behaviors flourish. The impression of a safe atmosphere is considered an essential prerequisite for FRC enhancement.

Additionally, the relationship between participative leadership and the advancement of followers’ creativity, particularly through the lenses of psychological safety and collaborative relationships, has become a focus point in modern academic discourse. Hoch et al. [20] stressed the importance of participative leadership in empowering people, which is critical for building a creative atmosphere. Frazier et al. [21] agree, arguing that psychological safety, a result of participatory leadership, is critical for encouraging risk-taking and innovative behaviors in firms. Furthermore, Oke et al. [22] found that collaborative ties were important in increasing creative problem-solving under participatory leadership. These findings are consistent with the findings of Carmeli et al. [23], who discovered a substantial link between leadership styles that prioritize employee participation and the stimulation of radical innovation. These studies highlight the profound effect of participatory leadership in building an organizational culture of innovation and creativity. Likewise, there is a scarcity of studies investigating the direct correlation between PL and FPS, suggesting the need for additional study. Nevertheless, prior studies have presented valuable insights into both CR and the creativity of followers.

Prior studies have examined the relationship between PL and employee creativity, but the results have been inconsistent. Some studies have found a negative association [3, 9, 14, 20], while others have found a significant positive association [18, 22]. Additionally, there are studies that have found no connection between the two factors [23]. Furthermore, there is a scarcity of studies that examines the direct impact of PL on FRC. Moreover, there is a scarcity of studies investigating the direct correlation between PL and FPS, highlighting the need for additional study. In addition, prior studies have shed light on FPS and employee creativity, but this study examines how and when CR may simultaneously affect radical creativity in followers [21], employed CR as an antecedent of FPS, however their findings contradicted our study. Fu et al. [7] examined how CR mediates employee creativity. However, they neglected boundary constraints that alter CR’s effect on follower radical creativity reactions. The request is for “more examinations of the CR for radical creativity in various contexts.

According to SIP theory, this study indicates that collaborative relationships (CR) facilitate FRC. By fostering high-quality relationships between followers and leaders, the leaders may encourage cooperation among their followers. Participatory leaders prioritize providing informative and useful feedback to their followers [5, 11, 21]. After bonding with their followers, they create a psychologically safe environment. Due to these consultations and perspective tactics, followers will get valuable information and abilities that boost creativity [22]. A CR includes foundational values and leadership that encourage participation and followers. Teamwork between leaders and followers fosters cognitive and behavioral appreciation, confidence, and loyalty. It builds a good leader-follower connection [23]. Followers feel psychologically comfortable and productive while working together. Increased understanding, knowledge, and views result from reciprocal resource exchange [19]. So, inventiveness increases. Risks and unique, safe, cooperative notions are more likely to be accepted. People may overcome anxiety and fear of failure in a collaborative setting, encouraging innovation and ingenuity [18]. Therefore, based on the abovementioned discussion, the present study assumes “collaborative relationships” as a moderating role between FPS and FRC. In accordance, this research plays an important role in fulfilling the contextual and knowledge gaps by associating the PL, FPS, CR and FRC.

However, there is still an opportunity to contribute to the existing literature where this study can help to add significant insights through investigating the link between participative leadership (PL) and followers’ radical creativity (FRC) through the application of the social information processing (SIP) theory and the mediating effect of follower’s psychological safety (FPS) in the association between participative leadership and FRC. In addition, collaborative relationship (CR) functioned as a moderator in the connection between FPS and FRC. Specifically, this study aims to address the underlying research questions.

### RQ

How do participative leadership and follower’s psychological safety have relationships with followers’ radical creativity including the moderating effect of collaborative relationship?

The concept of FPS refers to the ability to freely express creative ideas without being hindered by concerns of interpersonal risks or critiques that may challenge the existing social order. Besides, the phenomenon of FPS plays a crucial role in providing essential socio-cognitive support for fostering individual creativity. Therefore, the variable FPS has a mediating effect on the relationship between PL and FRC. The third objective of our study was to assess the potential impact of the quality of interactions between leaders and followers on the relationship between PL ties and CR. This research makes significant contributions to the existing body of knowledge. This study aims to examine the impact of PL on individual-level FRC while expanding upon past research that has mostly focused on team or individual innovation [12].

Manufacturing industry play an important role in the economy of Bangladesh due in part to their rapid development both in quantity and quality. Manufacturing industries make a significant contribution in representing 24.95% contribution to the gross domestic product (GDP) every year [19]. The primary factors for choosing Bangladesh’s manufacturing sector are the significance of fostering follower’s radical creativity and follower’s organizational innovation, as well as ensuring sustainability and long-term survival in a competitive economy [22]. Additionally, it is appealing due to its presence in both domestic and international markets, as well as its total contribution to the economy. Following this trend, the significance of follower’s radical creativity has been increasing, demanding manufacturing industry can create and sustain their competitive advantage by improving their innovation capability [18, 21].

Accordingly, this research contributes to PL and creativity in recent literature by investigating the relationship between PL and FRC. However, participative leadership may be particularly relevant in increasing the FRC, which has been neglected in the prior shreds of evidence [10]. Secondly, this research further advances the literature by investigating the mediating effect of FPS as a mediating factor between PL and FRC to identify, help and develop the proper leadership style and examine dynamic, complex intervening mechanisms for the manufacturing industries. An exploration of the theoretical underpinnings of the relationship between PL and individual-level creativity offers new insights into this important phenomenon. Thirdly, this study proposes collaborative relationships as boundary conditioning that can strengthen the effects of PL on nurturing the FPS and FRC. The interplay between two distinct leadership properties in shaping individual-level FRC can offer theoretical and practical insights. Finally, this research extends the SIP theory to employee FRC by demonstrating how FPS and collaborative relationships influence the leader-follower creativity relationship, leading to FRC [18] because FRC and innovation are required to survive and compete in today’s market [13]. The following section discusses the literature, proposes theoretical background and hypotheses development, clarifies the methodology adopted in this study, results and discussions with theoretical and practical implications and presents the key research conclusions. In addition to that, the perceived study drawbacks of the research are provided with future research suggestions and recommendations.

The remaining sections of this paper are organized as follows: Sect. 2 reviews the existing literature pertaining to our work. Outline of Sect. 3: Details data sources and methodologies, the empirical results are presented and discussed in Sect. 4, followed by a discussion (Sect. 5), final observations and conclusions in Sect. 6.

## Literature review

### Conceptual framework

Based on recent empirical studies, PL has been an essential factor influencing organizations’ creativity and innovation [29, 30]. PL is a humanistic leadership style where leaders involve subordinates’ suggestions, ideas, and opinions in decision-making. Moreover, these leaders are essential in promoting innovation in organizations [14] because they encourage intrinsic motivation, facilitate problem-solving, encourage a positive team climate, and provide support. Employees who are creative at work develop new ideas potentially useful for improving or renewing products, services, processes, and procedures [31]. According to Qu et al. [32], organizations possess the ability to adjust and respond to both internal and external changes. The relationship between PL and creativity has been the subject of prior studies, with inconsistent findings. Meta-analytic studies have shown a diverse range of associations between PL and creativity. Similarly, the inconclusive findings of Nabi and Liu [1] led scholars in the field of creativity to reevaluate the relationship between PL and follower creativity, specifically exploring the mechanisms via which PL may influence followers to engage in innovative activities. Previous studies have shown that colleagues who possess creative attributes, organizational characteristics, and external drives have a more pronounced practical impact on explosive innovation compared to other determinants. Scholars have posited that leadership within the textile and garment sectors has a noteworthy role in fostering innovative thinking, originality, and innovation of followers, particularly in industrialized nations [1, 10]. Given the transformative nature of the industry, organizational leaders are placing emphasis on the profound creativity exhibited by their subordinates. This emphasis is driven by the recognition that radical creativity is becoming increasingly reliant on factors such as employee willingness to take risks, dynamic leadership, creative aptitude, and extrinsic motivators, as highlighted by Fu et al. [7] and Jin and Qing [14]. Moreover, a primary objective of leadership is inspiring others to engage in actions that effectively contribute to the attainment of organizational objectives. As a result, past creativity literature supports FPS for engaging in creativity, which is the primary component of PL [33]. As a result, this investigation is novel in PL and FRC. However, the present research indicates that if CR is higher, tending to weaken the relationship.

### Underlying theory

The present study applies the social information processing theory (SIPT) in its model. The SIPT represents an elucidation of how communication (CMC) among various groups of people, sharing views, ideas, instruction, and command, all facilitate the creation of interpersonal connections. When initiating connections among multiple groups of people, there needs to be more communication indications, and impressions are mostly established solely on verbal and other modes of communication. The preset study indicated the communication among two groups of people, i.e., leader-follower or manager-subordinate relationship and communication, interaction, followership, reaction, leadership, and other relationship managing factors. The present research applies SIP theory to organizational leadership research has been established and validated by prior scholars [for example, 34, 35]. In circumstances of organizational settings, the concept of SIP theory posits that workplace attitudes and behaviors are shaped by the manner in which people establish connections, exchange information, and interpret their work settings.

Research on organizational leadership (for example [25, 36], has shown that employee creativity is facilitated by cognitive processes that are focused on creativity. These processes enable individuals to develop ideas and behaviors that are both original and useful and that correspond with creative notions. Leaders provide insights into the corporate climate, including the actions of creative people, which influence or shape employees’ perceptions of the environment [37]. Subsequently, employees proceed to exchange their respective perspectives about the aspect of safety in relation to undertaking risks. Participatory leaders exhibit a strong regard for employee input by properly acknowledging and valuing employee interests. As a result, workers experience a sense of acknowledgement and develop a collective understanding of security, leading to increased levels of engagement and a heightened inclination towards engaging in innovative endeavors. In accordance with prevailing patterns, our study demonstrates that the cognitive state known as FPS serves as a mediator in the association between Pl and FRC.

Nevertheless, there is a lack of empirical research that investigates the impact of FPS on the creative process and cognitive abilities of individuals, specifically in relation to FRC [38]. Despite extensive study, there are still gaps in theory development, notably concerning the complex paths via which participatory leadership influences radical creativity. The multidimensional character of psychological safety, as well as its interaction with other variables such as trust, motivation, and individual proclivity for creativity, necessitates further exploration. Moreover, the precise nature of ‘collaborative connections’ within the context of participatory leadership, and how these ties especially contribute to radical creativity, is unknown. While it is known that collaborative workplaces foster innovation, the methods by which they interact with leadership styles and psychological safety require further investigation.

### Hypothesis development

Several research studies have explored and evaluated the theory that links participatory leadership (PL) with followers’ radical creativity (FRC), highlighting the importance of psychological safety and collaborative partnerships. Somech [24] and Zhang and Bartol [25] found that participatory leadership positively impacts followers’ radical creativity. The underlying logic is that PL cultivates an environment where followers feel empowered and secure to take risks and voice new ideas. Individual creative skills for exploration can be developed through training in creativity and innovation [26]. However, there are some conflicts in the research findings. For example, while some research confirms that PL has a beneficial impact on creativity [23], others imply that this link may be influenced by factors like corporate culture, personality characteristics among followers, and the nature of the tasks [27]. The efficacy of PL in improving FRC may differ among cultural settings, and employee personal factors might modify this connection [28].

### Participative leadership (PL) and followers’ psychological safety (FPS)

PL describes the perception that ‘people are comfortable being themselves [7] and ‘feel able to show and employ themselves without fear of negative consequences to self-image, status or career [39]. Participative leaders are instrumental at large [10]. Accordingly, leaders can create an effective working social atmosphere that inspires and enhances creativity. Specifically, participative leaders cultivate a climate of FPS where followers are encouraged to take interpersonal risks and express themselves to realize their potential and grow [7]. PL strives to improve followers’ involvement by giving them more freedom, respect, authority, support, knowledge, and other resources. It also addresses problem-solving issues with followers by considering them before making decisions [40]. When the followers feel safe, their contribution to the organization will be higher [7]. Psychologically safe and healthy environments allow people to investigate cognitive pathways to come up with new and valuable ideas, identify challenges, collect data, and develop and assess solutions. As a result, followers feel inspired, supported, and responsible for generating creative solutions when participatory leaders involve them in collaborative decision-making and problem-solving [16]. Likewise, by exchanging ideas and receiving assistance, employees obtain valuable knowledge that can then be improved to create practical solutions. Despite the absence of prior empirical investigations examining the association between PL and FPS, Wang et al. [41] identified a significant connection with PL. Moreover, empirical analysis has yet to examine how PL empowers the follower’s FPS.

According to Chen [10], the theory of SIP explains that individuals acquire knowledge about the cognitive and behavioral reactions of others via their social interactions, thereby influencing their information processing. Thai is why we argue that PL is the “most humanistic approach to leadership” because it involves people in corporate decision-making, resulting in FPS. Follower–leader relationships make workers feels secure, embracing risks and questioning to alter upcoming challenges. Followers will be keener to seek thoughts and suggestions from co-workers and others inside and beyond departments if they are not afraid of unfavorable criticism. Second, PL emphasizes group decision-making. Based on Fu et al. [7], the technique ensures the harmonic decision-making process and promotes compliance among followers with given instructions. Employees who are actively involved in making decisions spend more time seeking and encoding information and coming up with better, more creative solutions to work difficulties, fostering an environment conducive to psychological well-being and the free flow of ideas.

According to SIP theory, employees construct views of their work environment based on feedback from leaders [42]. Participatory leadership methods, such as inclusive decision-making and recognizing employee contributions, may dramatically improve followers’ psychological safety by creating an environment in which people feel valued, respected, and free to share their views and concerns [43]. This interaction demonstrates how leadership style may influence employee behavior and attitudes by shaping the psychological components of the workplace [43].

#### H1

*There is a positive correlation between participative leadership and the psychological safety of followers.*

### Followers’ psychological safety (FPS) and followers’ radical creativity (FRC)

According to Social Information Processing (SIP) theory, Followers’ Psychological Safety (FPS) is critical for developing Followers’ Radical Creativity (FRC). Psychological safety promotes risk-taking and inventive thinking, both of which are necessary for creativity [19]. According to SIP, leaders’ behaviors considerably impact this psychological climate [44]. Kim et al. [39] described the term “psychological safety” as a work environment in which employees feel comfortable taking risks with one another. Perceiving and feeling high levels of interpersonal trust are just part of what it means to work in an environment where employees feel psychologically secure [33]. Pertinently, Hu et al. [27] found that people easily expose their disagreements. A previous study has found that FPS boosts creativity [21], but most studies concentrated on social contexts [36]. Prior research shows that FRC generates unique, original, and valuable ideas for goods, services, and processes. Follower’s creative ideas primarily result from cognitive and motivational processes within the individual and interpersonal collaboration among leaders and followers [10]. SIP theory suggests that psychologically healthy environments are necessary because creativity and innovation are unpredictable and hazardous. Therefore, FPS offers the necessary socio-cognitive underpinning for follower’s creativity enhancement and development [7].

Accordingly, psychologically secure and healthy companies can communicate creative notions and thoughts without fearing interpersonal hazards or condemnation for challenging the status quo [21]. Followers who feel comfortable psychologically are more likely to collaborate and cooperate without being interrupted by interpersonal conflict and challenges [10]. Moreover, when employees sense FPS, they might engage in risk-taking and proactive attitudes/behaviours in the workplace. Thus, followers who speak out or raise an issue are more likely to offer creative proposals for change and suggest adjustments to conventional processes, even when others disagree. Furthermore, according to researcher findings, followers’ creativity is strongly linked to FPS [45]. Apart from that, shared views of a psychologically secure environment boost followers’ creativity. Without fear of manipulation, followers express natural treatment, respect, and support for one another in their creative endeavors, enhancing the probability of FRC. Therefore, we hypothesized as follows:

#### H2

*Follower’s Psychological safety is positively related to followers’ radical creativity.*

### Mediating role of followers’ psychological safety

According to the Social Information Processing (SIP) theory, the mediating role of Followers’ Psychological Safety (FPS) highlights that employees’ perceptions of safety, shaped by organizational cues and leadership behaviors, have a significant impact on their work engagement and innovation [19, 21]. This psychological safety, developed in a supportive atmosphere, increases their willingness to voice ideas and take chances, both of which are necessary for organizational success [44]. Scholars (for example [22, 36], conceptualized psychological safety as the state in which individuals are able to freely express and use their attributes and abilities without apprehension about potential adverse outcomes that may impact their self-perception, social standing, or professional trajectory. According to Nabi and Liu [1] symbolization pertains to the establishment of a safe environment characterized by trust and mutual respect. This environment fosters a sense of comfort among followers, enabling them to expose their thoughts and provide novel ideas freely. Importantly, this environment also assures individuals that any errors or misconceptions will be acknowledged and embraced. However, FPS is crucial in influencing and assessing such risks and hazards. Creativity is not always encouraged and nurtured [3]. There may be resistance since organizations must finally face the repercussions if the intended goals are not met. When workers perceive a heightened degree of FPS, they are less inclined to evaluate the negative consequences of engaging in voice behavior and instead foster a feeling of commitment that contributes to the establishment of a supportive work atmosphere, thus promoting creativity. Accordingly, those who possess a sense of ease in establishing trust-based relationships and lack apprehension over potential consequences for failure are more inclined to engage in the generation of novel concepts [1, 3, 5]. A successful leader cultivates a work environment that exhibits a leader-member exchange of connections marked by high quality [7]. Additionally, they encourage workers to engage in divergent thinking and foster the establishment of trust.

In addition to that, FPS is a proximate mechanism linking PL and creative process engagement through the collaborative relationship (Wang et al., 2016). Hence, sound energy is generated by shared beliefs of safety. They are psychologically and cognitively aroused to recognize, seek out, and develop creative and innovative solutions to organizational challenges [3]. In an atmosphere that fosters FPS, workers have a sense of ease and confidence in expressing their thoughts and ideas, particularly those pertaining to new proposals about goods and processes, which are essential to the development of creativity. Therefore, collective ideas of safety provide followers with a sense of optimism. Individuals are motivated both emotionally and intellectually to actively recognize, explore, and create novel solutions for issues inside an organization. Thus, we put up the following hypothesis:

H3*Followers’ psychological safety mediates between the association of participative leadership and radical creativity of followers.*

### Participative leadership (PL) and followers’ radical creativity (FRC)

According to Social Information Processing (SIP) theory, PL fosters a supportive environment by encouraging open communication and idea exchange [5]. PL increases intrinsic motivation among followers by including them in decision-making [46]. In accordance with Chen [10], participative leaders engage in information sharing, encourage the advancement of innovative ideas, and motivate people to go beyond conventional boundaries in order to achieve success. On the other hand, Shao et al. [47] extensively analyzed the concept of PL and have shown that it effectively improves performance objectives for subordinates and transforms their beliefs and self-perception towards greater aspirations. Positive follower outcomes, including employment satisfaction, innovation and work involvement, success at work, and organizational retention, have been shown to be associated with PL [9]. Recently, there has been a significant increase in the study of PL, with a particular focus on moving from idealized representations to more concrete conceptualizations. This trend has been seen and documented by researchers such as Shao et al. [44] and Wang et al. [33]. The results of this investigation, on the other hand, exhibit contradictory and puzzling outcomes. Further investigation is required because of the dynamic relationship between PL and the creativity of its adherents. Thus, PL refers to a management approach whereby leaders actively encourage and engage workers in the processes of decision-making and problem-solving. Based on the SIP theory, it is posited that followers tend to value and support the practice of seeking consultations rather than receiving explicit orders and directives [1, 10]. Pertinently, Gazi et al. [48] suggested that people have a sense of psychological safety and empowerment within the context of PL. It has been shown to have a positive impact on employee engagement, self-motivation, commitment to the company, and citizenship behaviors in organizations. Moreover, Chen [10] found that PL is able to reduce anxiety levels, promote active involvement of subordinates in the creative process, and cultivates a climate conducive to creativity.

Similarly, participative leaders believe in establishing and maintaining a collaborative environment by applying a participative and reward system for employees [10]. According to Cascini et al. [4], there is a preference for open communication during interactions with followers, emphasizing the importance of honesty and candor. This approach aims to foster mutual trust and create a psychologically comfortable atmosphere. Moreover, by considering employee interests, participative leaders signal that employee input is valued and appreciated. Employees feel recognized and share perceptions of safety, so they become more engaged and pursue creative activities. According to SIP theory, individuals acquire knowledge about others’ cognitive and behavioral reactions through social and interpersonal relationships, subsequently influencing their information processing [49]. Consequently, we propose that PL is the “most humanistic approach to leadership” by engaging individuals in organizational decision-making, which leads to FPS [10].

Firstly, participative leaders motivate workers to exchange knowledge, identify problems, discover opportunities for improvement, create new ideas, and offer appropriate alternatives for promoting FRC [50]. Moreover, information exchanges serve as conduits for comprehending and appreciating creative thinking processes and behaviors. A psychologically complex free environment and collaborative information sharing between leaders and followers ensure the creativity enhancement propensity of followers. Secondly, creativity naturally includes difficulties, uncertainty, and risk because fresh ideas are not promised to produce the intended results [49]. Furthermore, sometimes, new ideas created by followers may need to be welcomed or recognized by their leaders. Hence, the establishment of a conducive work environment that facilitates the undertaking of interpersonal risks and the generation of novel ideas is crucial for fostering the radical creativity of followers.

#### H4

*Participative leadership is positively associated with followers’ radical creativity.*

### Moderating role of collaborative relationship (CR)

Within the framework of Social Information Processing (SIP) theory, the Moderating Role of Collaborative Relationships (CR) shows that CRs moderate how individuals interpret social cues and information in the workplace, influencing their behavior and attitudes [51]. This moderation is critical in situations where team collaboration and joint problem-solving are required, as it allows for a better understanding and integration of varied perspectives [52]. Information sharing is important for maintaining good relationships. In this process, one party transfers its information or knowledge to others [46]. Sharing information between two parties is necessary for learning in a relationship [41]. Collaborative relationships are agreements and acts by cooperating groups to share resources to achieve a common purpose [28]. Collaboration requires the participation and cooperation of at least two parties that agree to share resources such as money, information, and people [53]. Organizations in a collaborative partnership have shared goals and objectives. The goal of a collaborative relationship is for all parties to gain from working together [53]. To establish a collaborative relationship between leader-follower, interaction and exchange of information and knowledge must foster creativity (Wang et al., 2022). Collaboration and leader-member interaction will enhance FPS and followers’ psychological empowerment for generating ideas and fostering creativity [7]. On the other hand, Frazier et al. [21] claimed that the creativity of followers can be affected by cognitive concepts related to autonomy, FPS, and behavioral engagement in creative endeavors. Besides, Fu et al. [7] indicated effectiveness and efficiency are connected to leader-followers’ activities, transmit, integrate, and use information are vital factors in collaborative relationships.

According to SIP theory, collaborative interactions have a favorable correlation with FRC. Leaders may promote collaboration among followers by fostering high-quality interactions between followers, as well as between leaders and followers, and among leaders themselves. Participatory leaders prioritize expressing their opinions comprehensively and helpfully to their followers. They do so after establishing strong connections with their followers, ensuring that the setting is psychologically secure. Followers will obtain vital knowledge and skills via these beneficial consultations and perspective techniques, enabling them to attain higher levels of creativity. Moreover, a collaborative connection encompasses the fundamental principles and the collaboration between leaders who encourage participation and their followers. A collaborative connection between leaders and followers promotes the development of cognitive and behavioral appreciation, confidence, and loyalty among followers. Additionally, this connection helps to construct a high-quality relationship between leaders and followers. The collaborative connection fosters a sense of psychological safety among followers, motivating them to exert more creative efforts. It also facilitates the exchange of reciprocal resources, leading to increased understanding, knowledge, and the sharing of perspectives. Consequently, this enhances overall creativity. Ultimately, people are more likely to embrace risks and go into novel, safe, cooperative concepts. A collaborative atmosphere fosters an environment where people may overcome anxiety and fear of failure, developing behaviors that encourage creativity and resourcefulness [21]. To summarize, the underlying moderation relationships are hypothesized:

#### H5

*Collaborative relationship has a moderating effect between followers’ psychological safety and followers’ radical creativity.*

Thus, the proposed conceptual framework for this study is as follows:

## Methods

### Target population and unit of analysis

**Fig. 1:**
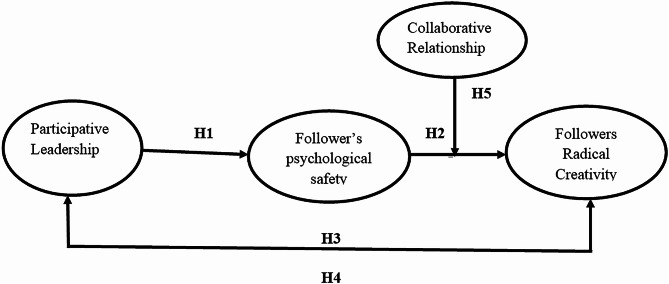
The proposed conceptual research framework

This research gathered data from Bangladeshi manufacturing industries using a survey questionnaire method. The target populations in this study are the managers who are working in the ready-made garments, pharmaceuticals, telecommunications, constructions, and agro-food processing industries under the R&D, HR, and process engineering departments. The selection of the manufacturing sector was based on its significant role in fostering the economic development of Bangladesh. In Bangladesh, the manufacturing industry has emerged as the primary contributor to the country’s GDP, accounting for the largest proportion (88%) in 2021. Accordingly, the respondents were the leaders (top, mid and lower level), institutional experts, and followers. The research was conducted in many locations, including ‘Dhaka’, ‘Savar’, ‘Gazipur’, ‘Narayanganj’, ‘Narsingdi’, ‘Munshiganj’, and ‘Chittagong’, as well as in Export Processing Zones, i.e., EPZ; Bangladesh Export Processing Zones Authority (BEPZA), and Special Economic Zones (SEZ) (Fig. 1).

### Measurement of constructs

Pioneering organization research needs to comprehend how leadership systems influence creative end results in order to create adaptable innovative environments. The proper assessment method for leadership involvement and safety along with relationship quality and radical creativity within followers directly reveals their impact on organizational success. The measuring constructs used in this study include four essential aspects of organizational dynamics including participatory leadership and psychological safety and collaborative relationships and follower radical creativity. First, the paradigm developed by Al Wali et al. [54] is used to evaluate participatory leadership. It looks at leader-follower involvement in decision-making and includes metrics such follower satisfaction with participation, communication clarity regarding involvement, and frequency of consultations. Leaders and followers satisfaction improves due to participatory leadership which involves inclusive decision mechanisms that create better organizational results [90, 91]. Nabi and Akter’s [92] study presents participatory leadership through multiple dimensions which enables leaders to involve followers actively in decisions to enhance their workplace connection while driving Organizational goal attainment [93]. The inclusive strategy promotes dialogue along with team member belonging and this effect is strengthened through psychological safety principles. According to Edmondson (1999) psychological safety provides individuals with protection from fear which creates innovative environments where team members will take necessary interpersonal risks to express their creativity. Second, in accordance with Edmondson’s [55] research, the psychological safety of followers is assessed by taking into account markers such as perceived trust in expressing opinions, comfort in taking psychological risks, and supporting behaviors seen in teams. The foundation created by psychological safety according to Chen et al. [95] allows workers to safely take risks and share innovative concepts while preventing potential negative outcomes. Drawing on the work of Gazi et al., [56] and Berson et al. [57], the Collaborative Relationship construct emphasizes shared goals, mutual trust, commitment, and frequency of communication among collaborators. Essential to performance improvement and team cohesion development is building collaborative relationships because evidence shows mutual trust along with shared goals leads to better organizational results [94]. The fundamental element for successful teamwork and collaborative issue resolution stems from shared goals and reciprocal reliance connections between team members as identified by Kundi and Shahid [96] in collaborative relationships. Lastly, following the lead of Hair Jr et al. [58] and Fu et al. [59], Follower Radical Creativity evaluates the amount and caliber of original ideas, the degree of risk-taking in suggesting nontraditional solutions, and the creative actions or products that result from followers’ creative contributions. The organizational innovation depends heavily on follower radical creativity which expresses itself through original ideas and risky behavior. Research demonstrates organizations gain superior market adaptation by asking their followers to contribute creative solutions leading to outperforming competition [97]. Organizations achieve better creative output understanding through relationship measurements of their communication patterns and interpersonal dynamic structures. The innovative implementation of Follower Radical Creativity functions as an essential dimension which evaluates both the creative dimensions and the production quality of team member work [95, 98]. In the context of this study, these constructs [55, 57, 60–62]and [8] provide a thorough framework for examining organizational leadership, team dynamics, and creativity. The model emphasizes establishing a conducive environment for intellectual risks that simultaneously protects employees from harm during their processes of idea development. Organizations pursuing innovative growth need to focus on both the relational aspects and psychological dimensions of their procedures as these constructs demonstrate essential links between leadership and teamwork with creativity [92, 99].

### Questionnaire and scale development

With reference to the existing literature and validated scale, we have developed the research questionnaire. Two questionnaires were used: one for the followers and one for their immediate leaders. The first author and research assistant spoke to all the respondents (leader and follower groups individually) to brief them on the study’s aims and objectives, explain the survey processes and give them the questionnaire and a return envelope. Each questionnaire included a researcher-assigned ID number to connect followers’ replies with leader ratings. To preserve secrecy, respondents sealed completed surveys in envelopes and submitted them to researchers. The questionnaire was administered at the company’s conference and in a meeting room. The researcher promised to keep the information secret and confidential. As a pilot test of the questionnaires, the researcher solicited the opinions and perspectives of 21 business professionals, authorities, and legislators to appraise the reliability of the survey and govern how the informants interpreted the various survey parts.

Creativity, inventiveness, sustainability, and long-term survival in the competitive edge are the primary reasons for picking Bangladesh’s manufacturing sector. Furthermore, it is attractive because of its contribution to its economy, both domestically and internationally. The questionnaire was first produced in English, and then a competent and experienced professional translator from the industry, university, or academics translated it into the native language of the area, which was Bengali. The translated questionnaire was subjected to a comparison process in order to ascertain its substantial equivalence and accuracy. All items are effectively aligned using a five-point Likert scale ranging from “1 = Strongly Disagree” to “5 = Strongly Agree”. Table 1 presents a comprehensive overview of the demographic characteristics of the participants. Following this, we control demographic factors such as gender, age, education levels, and employment length that affect FRC. Age and employment duration were operationalized in terms of genuine years. Appendix A shows all the measurement items in detail.

### Data collection procedure

Printed copies of the questionnaires were sent to the directors of the manufacturing enterprises, accompanied by a cover letter that provided an overview of the study’s purposes, as well as its importance and potential ramifications. The survey questionnaires were sent to 129 leaders or managers and 260 followers (employees) by the researchers. In accordance, each leader is allocated a team consisting of 3 to 5 followers based on the nature of the organization. The answer to this question is contingent upon the specific characteristics and scale of the organization in question. After the survey completion, 92 leaders responded finally and 257 employees provided their responses as well. Consequently, a methodology based on simple random sampling was used in the selection of survey respondents. The researchers used Yamane’s method to rationalize the sample size that was collected, as recommended by Nabi and Liu [1]. We kept and set aside a few responses after modifying the answers and comments from the supervisor and followers. There were almost 27 incomplete, below standard, and not up to the mark responses, which were removed from the list of questionnaires. After removing incorrect and incomplete as well as below standard questionnaires, 322 questionnaires were useful. Thus, 89.72% of questionnaires were returned, while 7.74% were removed due to incomplete, non-matching, or poor-quality responses, yielding an 82.78% effective response rate. Consequently, the final response rate in this research was 82.78%, which was a satisfactory level of participation. Considering that previously Karim et al., [69] had a response rate of 79%, Shahneaz et al. [70] 77.9%, Amin et al. [71] 52.25%, Mahmud et al. [72] 47.2%, and Amin & Oláh [73] had a response rate of 41.8% in the context of Bangladesh. Besides, the survey data was collected from middle of the March to December 2021.

### Demographic information of the participants

The descriptive section illustrates the demographic profiles of survey participants who completed the survey questionnaire. The participants provided information about their sex (‘male,’ 1, ‘female’ 2), age (in years), education (1, ‘undergraduate,’ 2, ‘graduate,’ 3, ‘post-graduate 4, ‘Doctorate’), organizational work experience, Industry, and particular work-related department. Among the survey questionnaire, 322 responses were found useable. The effective response rate is 82.78%. Out of 322 participants who provided information about their sex, 287 (89.13%) were male and 35(10.87%) were female. From the perspective of age in years, 58.07% of participants were more than 24–36, 32.61% were 37–47 years old, and 9.32% were more than 48 years old, while the majority of the participants were 58.07%. In terms of educational qualifications of respondents, 7.04% of the Ph.D. holders, 27.64% were postgraduate, 54.03% of the respondents were undergraduate and 11.08% were the respondents of graduated. Considering work-related experience, it has been shown that experience ranges from highest to lowest 33.23 (6–11) and 10.25 (more than 22). In addition, the data collected from the Ready-Made-Garments industry is 63 (20.19%), the pharmaceuticals industry is 19 (22.98%), and the telecommunications industry is 32 (16.77%). Moreover, the construction industry collected 17 (20.81%), and the agri-foods processing industry represented data collection almost 47 (19.25%). In addition, 142 (44.10%) of participants have come from the research and development department while 117 (36.34%) respondents were from the human resource management department and 63 (19.57%) from the process engineering department. Table 1 provides the demographic information of the participants considering age, gender, education, experiences, and department.

**Table 1:** Demographic factors of the respondents

Variables	Values	Frequency(*N*)	Percentage%
Gender	Male	287	89.13
	Female	35	10.87
Age	24–36	187	58.07
	37–47	105	32.61
	> 48	30	9.32
Educations	Undergraduate	36	11.18
	Graduate	174	54.03
	Post Graduate	89	27.64
	Doctorate	23	7.14
Work experience	< 6 years	92	28.57
	6–11	107	33.23
	11–16	48	14.91
	17–22	42	13.04
	> 22	33	10.25
Manufacturing Industries	Ready-Made-Garments (RMG)	63	20.19
	Pharmaceuticals,	19	22.98
	Telecommunications,	32	16.77
	Constructions	17	20.81
	Agri-Foods Processing	47	19.25
Department	R & D	142	44.10
	Human Resource	117	36.34
	Process Engineering	63	19.57

### Analysis tools

PLS-SEM is a statistical method that is used for estimating path models with latent variables. This methodology is considered to be multivariate and non-parametric in nature. The researchers use the PLS approach for structural equation modeling to evaluate the hypotheses [58, 61]. We used the PLS-SEM technique in this investigation since it was deemed appropriate for the exploratory character of the work [63]. Besides, PLS-SEM analysis technique involves the evaluation of both the measurement model and the structural model consecutively. The measurement model evaluates the factor loadings, AVE, convergent, and discriminant validity (FLC, cross-loadings, and HTMT). Moreover, the second stage is to undertake a “structural model” assessment to ascertain the hypothesized relationships among the proposed model latent components through assessing and evaluating the coefficient of determination (R2), effect Size (ƒ2), and predictive relevance (Q2). Moreover, the study used the importance-performance map analysis (IPMA) to assess the importance and performance of the exogenous constructs for FRC.

In this study, PLS-SEM analysis was used. PLS-SEM was preferred over covariance-based SEM because of its prediction-oriented statistical technique, which is resilient to non-normal data and small-sized samples [74, 75]. The covariance-based approach (CB-SEM) and the variance-based (VB-SEM) approach are typical methods for calculating SEM parameters. Urbach & Ahlemann [76] state that CB-SEM uses the maximum likelihood (ML) approach to minimize the difference between observed and predicted covariance. ML may be utilized if CB-SEM data distribution is normal. Chin & Newsted [77] found that 200 to 800 instances are needed for CB-SEM analysis accuracy and dependability. This work used VB-SEM to estimate model parameters from loadings and path values. The methodological goal was also to diminish the discrepancy between sample covariance and theoretical model predictions. VB-SEM also reduces the covariance matrix of observed measures’ appropriateness [76]. CV-SEM is ideal for confirmatory research and theory testing. Variance analysis is used in PLS-SEM, a structural equation modelling (SEM) method. Researchers often use PLS-SEM to anticipate and explain large target component change using numerous explanatory factors [74]. Instead of relying on covariance to explain item relationships, the PLS-SEM technique maximizes explained variation in the dependent variable via the independent variable. Smart-PLS was chosen over AMOS because it can simultaneously provide compound measurement model and structural model results [78]. Therefore, this research used PLS-SEM to analyze and interpret the data owing to its reliable predictions.

### Ethical guideline and consent of the participants

For this investigation involving human participants, an ethical review and authorization were acquired from the ethics committee in accordance with local legislation and institutional norms. The permission for this research was granted by the School of Management Human Research Ethics Committee of Juijiang University, China, which signifies that it has met the ethical requirements. Written informed consent was acquired from all individual participants for the collection, storage, and use of the information they provided for research purposes. Moreover, written consent was also collected from the supervisor or, manager or, superior authority of the participants. Responses were obtained without any compulsion, and all participation was entirely voluntary. Participants were provided with information on the goal of the research and the potential benefits before the questionnaire was posted or shared on social media. It was explicitly stated that volunteers would not be given any financial remuneration for their involvement. A platform for open discussion was made available to participants, allowing them to inquire and get a deeper understanding of the research. Additionally, participants were explicitly told of their option to withdraw from the study at any point. The processes were conducted in strict adherence to the applicable norms and legislations.

## Results

### Normality test

To determine whether or not each independent variable was normally distributed, we utilized the skewness-Kurtosis test as suggested by Hair et al. [61]. Researchers observed that their promising scales found encouraging and promising results, including findings on both investigated scales. Table 2 shows the skewness and kurtosis values ranging from − 2 to 2 and − 7 to7, assuming a standard distribution.

### Control of CMB

A survey-based questionnaire was used to gauge the validity of the research’s central hypothesis. Information for this research was gathered from various sources, primarily the Bangladeshi manufacturing sector. Because of this, common method variance may develop (CMV). Statistically, the datasets were verified using Harman’s single-factor technique. In the conducted investigation, it was shown that a singular component accounted for 33.2% of the observed variance, which is below the threshold of 50%. Additionally, the researchers used the statistical technique known as “Collinearity Statistics,” which produces “Variance Inflation Factors (VIFs).” Thus, common method variance (CMV) might potentially contaminate and distort the model’s results when the Variance in Inflation Factors reaches a threshold of five or above. The investigation has identified “Collinearity Statistics” with an anticipated count of five or less. The findings of this investigation, as shown in Table V, indicate the absence of common technique variance.

### Measurement model

The measurement modal employs confirmatory factor analysis to assess the level of fit quality of the study model that is provided. The evaluation of factor loading, discriminant validity (DV), Cronbach’s Alpha, composite reliability (CR) and AVE is conducted through the application of the Fornell-Larcker and Heterotrait-Monotrait Ratio (HTMT) Criteria, as suggested by Hair Jr et al. [58].

**Table 2:** Weights and measures of the construct loading development outcomes

Items	Standard Deviation	Kurtosis	Skewness	T-statistics	*P* Values	2.5%	97.5%	VIF
CR1	0.97	1.31	-1.19	110.27	0.000	0.33	0.35	2.70
CR2	0.98	1.36	-1.22	57.18	0.000	0.33	0.35	1.74
CR3	0.97	1.46	-1.45	95.23	0.000	0.33	0.34	1.29
FPS1	0.94	1.22	-1.78	17.68	0.000	0.26	0.33	1.70
FPS2	0.86	1.21	-0.99	13.71	0.000	0.24	0.31	4.96
FPS3	0.91	0.96	-1.13	18.56	0.000	0.25	0.31	1.71
FPS4	0.97	1.18	-1.14	10.95	0.000	0.11	0.16	1.31
FPS5	0.99	1.27	-1.82	11.36	0.000	0.11	0.16	1.70
FPS6	1.00	1.43	-1.26	11.52	0.000	0.11	0.16	1.89
FRC1	0.98	0.84	-1.94	20.33	0.000	0.20	0.24	1.88
FRC2	0.95	0.85	-1.67	22.99	0.000	0.23	0.27	1.82
FRC3	0.92	0.44	-0.93	24.07	0.000	0.23	0.27	1.61
FRC5	0.91	1.13	-1.01	22.64	0.000	0.21	0.25	2.72
FRC6	0.94	1.26	-1.10	22.13	0.000	0.26	0.31	2.84
PL1	0.99	0.88	-0.91	24.19	0.000	0.20	0.23	2.51
PL2	0.95	0.91	-0.67	23.52	0.000	0.23	0.28	2.10
PL3	0.92	0.79	-1.71	23.47	0.000	0.24	0.28	1.65
PL5	0.94	1.25	-1.08	21.09	0.000	0.27	0.33	2.10
PL6	1.00	0.95	-0.89	22.28	0.000	0.19	0.23	1.23

To assess the model’s accuracy and validity, two alternative methods were used. The first parameter is SRMR, and the second is NFI, which has a normed value index. The SRMR values were < 0,08 or < 0,10 to verify the suggested modal fitness and avoid side-by-side model misspecification, and NFI must be lower than 0.95 [64]. With SRMR = 0.0875 and NFI = 0.0931, our model satisfies the model fit criterion (Table 2).

The assessment of internal consistency, coherence, reliability, and exactness of the variables was conducted using Cronbach’s alpha, with a minimum threshold of 0.50. In order to assess the compatibility and robustness of the variables, the composite reliability (CR) must exceed a threshold of 0.70, as proposed by Fornell and Larcker [62]. The study ultimately identified Rho A and AVE as significant findings. The coefficient of Rho A exhibits a value of 0.70, indicating a need for a value greater than 0.70. Similarly, the AVE shows a value of 0.50, signifying a need for a value that is more statistically significant than 0.50. Table 3 provides the relevant information pertaining to the variables under consideration. It includes the CR, Cronbach’s Alpha, AVE, and the reliability coefficient rho A. Furthermore, the statistical analysis conducted on Table 3 generated several key findings. Specifically, the mean, standard deviation, correlation coefficient, and discriminant validity have been determined and are presented in the table. The values and their corresponding loadings derived from the remaining variables are presented in Table 4.

**Table 3:** Construct Reliability and Validity (*N* = 322)

Main Constructs	Items	Loadings	Cronbach’s Alpha	rho_A	Composite Reliability	Average Variance Extracted (AVE)
CR	CR1	0.94	0.90	0.91	0.91	0.67
	CR2	0.88				
	CR3	0.99				
FPS	FPS1	0.89	0.88	0.92	0.90	0.60
	FPS2	0.84				
	FPS3	0.77				
	FPS4	0.70				
	FPS5	0.72				
	FPS6	0.71				
FRC	FRC1	0.71	0.87	0.87	0.90	0.65
	FRC2	0.81				
	FRC3	0.83				
	FRC5	0.83				
	FRC6	0.86				
PL	PL1	0.78	0.86	0.87	0.90	0.65
	PL2	0.81				
	PL3	0.81				
	PL5	0.84				
	PL6	0.78				

Note: Using Bootstrap with 5000 people, *p* < 0.10, *p < 0.05, **p < 0.01, ***p < 0.001

CR = Collaborative Relationship, FPS = Followers Psychological Safety, FRC = Followers Radical Creativity, PL = Participative Leadership

**Table 4:** Discriminant validity, correlation coefficients, mean, and standard deviation

	Fornell-Larcker Criterion					Heterotrait-Monotrait Ratio (HTMT)				
	Mean	S. D	Age	Gender	Education	Experience	CR	FPS	FRC	PL
Age	3.811	1.391								
Gender	1.106	0.307								
Education	3.336	0.679								
Experience	1.401	0.629								
CR	4.383	0.972	0.85							
FPS	3.934	0.958	0.71	0.83			0.56			
FRC	3.902	0.946	0.40	0.56	0.81		0.42	0.77		
PL	3.887	0.956	0.40	0.44	0.58	0.80	0.42	0.65	0.56	-

Notes for Table 4; *N* = 322; Using Bootstrap with 5000 people, *p* < 0.10, *p < 0.05, **p < 0.01, ***p < 0.001; CR = Collaborative Relationship, FRS = Followers Psychological Safety, FRC = Followers Radical Creativity, PL = Participative Leadership

Furthermore, it is crucial to establish a higher level of discriminant validity, as measured by the AVE, compared to the level of validity established for the variables themselves. It ensures and upholds internal congruence, consistency, and validity in the associations among constructs [62]. The study results indicate that discriminant validity, as shown in Table 4, is of significant value. The use of HTMT (Heterotrait-Monotrait Ratio) in the research was employed to assess Discriminant validity and confirm the enhanced reliability and validity of the proposed components.

### Structural model

The measuring model was assessed before our investigation and yielded a statistically significant outcome. The verification and examination of the structural modal will now be conducted. Table 5 presents a comprehensive depiction of the overall, indirect, and direct effects of PL, the PS of followers, and the impact of collaborative connections between followers and leaders in facilitating FRC. Researchers and investigators would use the Path coefficient (β), T-statistics, and P-Values to determine if the hypothesis is significant or not. Table 5 shows that PL substantially affects followers’ FPS (β = 0.85; T = 66.42; *P* < 0.001). Therefore, H1 is significant and supported the proposition. Similarly, when followers feel psychologically safe, not worrying about their ideation and conception, new and novel ideas are created, and the radical capability of followers is increased. Thus, followers’ FPS considerably generates and promotes FRC among followers (β = 0.84; T = 5.66; *P* < 0.001).

Therefore, Hypothesis 2 is supported. Moreover, FPS significantly influences PL and FRC among followers (β = 0.25; T = 5.67; *P* < 0.001), proving that H3 is supported significantly.

Moreover, results indicate that PL has a significant positive impact on FRC (β = 0.78; T = 22.36; *P* < 0.001) through boosting followers’ autonomy, confidence, and trust, and therefore the H4 is significant. Conversely, the moderating role of the CR of followers-leaders in between the FPS and FRC is insignificant (β = 0.29; T = 0.66; *P* > 0.51). Thus, H5 is not supported. Detailed information on Tables 2 and 5 is included in the research.

Furthermore, results indicate that PL has a significant impact on FRC (β = 0.78; T = 22.36; *P* < 0.001) through boosting followers’ autonomy, confidence, and trust, and therefore the H4 is significant. Conversely, the moderating role of the CR of followers-leaders in between the FPS and FRC is insignificant (β = 0.29; T = 0.66; *P* > 0.51). Thus, H5 is not supported. Detailed information on Tables 2 and 5 is included in the research.

### Mediation of followers’ psychological safety (FPS)

According to the criteria and recommendations of Rahi et al. [65], the research is carried out to empirically investigate the mediating mechanism of the linkages of constructs. Both the lower and upper limits of the biased confidence interval (CI) are determined by the indirect effect [65]. The presence of a complete mediation effect is shown when the bias-corrected confidence interval (CI) of the bootstrap analysis encompass higher and lower than zero, i.e., both positive and negative values. The 0.05 level is used to determine the significance level. The results of the bootstrap indicate the indirect impact of PL -> FPS -> FRC, as seen in Table 5, (β = 0.78*0.84 = 0.6552, t-values of 5.67, p-values 0.000, SE = 0.000124) was entirely significant at *p* < 0.002. The indirect effects of 0.6552 shows that the bias-corrected bootstrap confidence interval (LL = 0.16, UL = 0.33) does not represent zero. These results showed that FPS mediation has a significant indirect effect on promoting FRC among followers. As a result, the results indicate that FPS is a mediator in the relationship between PL and FRC.

### Moderation of collaborative relationship

The suggestions put out by Hair et al. [58] were acknowledged and embraced in order to examine the moderating effect of the interaction between the different constructs. In accordance with the suggestion of Hair et al. [61], while we run the bootstrapping in PLS-SEM, the findings demonstrate that the lower and upper confidence intervals do not exhibit convergence towards zero or a proximate value of zero, it can be concluded that the confidence intervals lack reliability. Regardless of the magnitude of moderation, the outcomes and deductions will invariably uphold the perspective. When the value surpasses the threshold of zero, it adopts an inflexible stance and adamantly rejects the notion of temperance. The findings from the bootstrapping analysis are shown in Table 5. The results represent that the indirect impact of moderation on the link between PL > CR-> FRC (β = 0.29, T-values of 0.66, SE = 0.0000031) was found to be statistically insignificant at a significance level of *P* < 0.051. The bias-corrected bootstrap confidence interval (LL=-0.02, UL = 0.01) indicates that the indirect impact of the variable is 0.29, which is almost negligible. The moderation of corporate responsibility (CR) is seen to be inconsequential and lacks substantiation for the concept.

**Table 5:** Path analysis of follower’s radical creativity

Effect	Path	β	t-statistics	*p*-values	SE	Bias Corrected CI 2.5% 7.5%	
Direct effect	CR -> FRC	-0.12	4.17	0.00	0.000393	-0.18	-0.07
	FPS-> FRC	0.29	5.66	0.00	0.000255	0.19	0.39
	PL-> FPS	0.85	66.42	0.00	0.000331	0.82	0.87
	PL-> FRC	0.78	22.36	0.00	0.000293	0.71	0.85
Indirect Effect	PL -> FPS -> FRC	0.25	5.67	0.00	0.000224	0.16	0.33
Total effect	CR-> FRC	-0.12	4.45	0.00	0.000293	-0.19	-0.07
	FPS-FRC -> FRC	0.29	0.66	0.510	0.000231	-0.02	0.01
	FPS -> FRC	0.84	5.64	0.00	0.000155	0.19	0.39
	PL -> FPS	0.78	67.01	0.00	0.000231	0.82	0.87
	PL-> FRC	-0.12	22.59	0.00	0.000393	0.71	0.85

## Discussions

In rapidly developing economies like Bangladesh, industrial companies expect leaders who possess not just front-line leadership skills but also the ability to foster a participatory atmosphere, provide autonomy and independence, demonstrate visionary talents, and cultivate FPS culture. This research investigates the potential of PL to promote CR. Eventually, it advocates for FRC in light of desired work results, which is considered essential in the current corporate landscape for attaining sustained competitive advantages. This research aims to contribute to the existing body of literature on the manufacturing industry by examining and establishing the correlation between PL and FRC in the manufacturing sector of Bangladesh. The exploratory nature of this study has theoretical ramifications.

Our study contributes to the existing literature on PL by introducing and empirically examining a new conceptual framework. This framework proposes that PL influences FPS, which in turn affects FRC, with the moderating role of CR. In contrast to other studies on the subject, this study expands the scope of SIPT to include both FPS and CR. In addition, it examines the connection between PL and FRC, contributing to the existing body of literature on the dynamics of the leader-follower relationship. However, the present research knows little about the underlying mechanics of PL, even though it has been shown to influence employee outcomes [6, 8]and [25]. Consequently, we extend our findings by emphasizing the significance of FPS environments for followers and CR intervening mechanisms in the PL-FRC paradigm [36]. Consequently, the conceptual model underwent empirical testing and validation, revealing a direct and indirect relationship between PL and FRC. The results also suggest that PL develops the creativity of followers and improves the overall creative work environment. There is a prevailing belief that the presence of creative individuals is essential for achieving long-term sustainable competitive advantage in the manufacturing sector, particularly in light of the intensifying competition. Industry leaders must be fully aware and knowledgeable about the association between FRC and PL. Furthermore, leaders in the manufacturing industry need to adopt a participative leadership style in order to cultivate and augment the creative abilities of their subordinates. In addition, they possess the ability to impart creative thinking processes to their adherents that are vital to the cultivation of creativity. These skills are expected to enhance the observational and technical capabilities of individuals in order to achieve expertise in the field of manufacturing systems and engineering. As stated by Cascini [65], PL has a substantial impact on followers’ creative talents by creating a work climate that encourages creativity and open communication. According to this study, when leaders engage in participative practices, they transmit critical social cues that alter their employees’ cognitive frameworks and creative processes [5]. These empirical results support previous studies on participative leadership and followers radical creativity [11, 19, 26, 69, 76].

Secondly, this research elucidates the notable and affirmative correlation between PL and FPS. Additionally, it is crucial for workers to possess a heightened degree of FPS since this attribute is well-recognized as a significant factor in the production of innovative and imaginative outcomes. The results of the study also indicate that enhancing FPS may have a positive impact on their creative abilities in effectively completing given tasks. Similarly, this work has made a valuable contribution to the existing body of literature by effectively combining the principles of PL and FPS. For instance, Kim et al., [39] discovered that PL considerably improves FPS because inclusive decision-making generates an environment in which employees feel valued and safe. Similarly, Wang et al. [41] revealed that PL practices connect favorably with FPS, emphasizing that this leadership style minimizes workplace fear and facilitates open communication. This research supports the good influence of PL in establishing a psychologically secure workplace. The findings of this study specifically emphasize the involvement of participative leaders in employee decision-making processes. These leaders actively encourage employees to cultivate a sense of ownership, identify organizational issues, actively seek solutions, generate innovative and valuable ideas, and propose creative alternatives. The existing body of research examining the association between FPS and creativity has produced inconclusive results [56]. This study suggests that although FPS has often been identified as a factor that might predict creativity, its influence on this connection is also influenced by other factors, leading to modifications in the expected outcomes.

Thirdly, it is seen that FPS plays a significant role in mediating the association between PL and FRC. The findings of this study further contribute to the existing body of research by elucidating the mediating function of FPS in the relationship between PL and FRC, as previously identified by Gazi et al. [48]. The examination of the indirect influence of CR on FRC with the mediation of FPS is an additional scholarly contribution to the existing body of knowledge on the subject of CR. Al Wali et al. [54] showed in their key study that FPS strongly mediates the influence of PL on FRC, implying that when employees feel secure taking chances without fear of negative repercussions, they display higher levels of creativity. Similarly, Ethics [60] discovered that PL improves FPS, which creates an environment conducive to radical innovation among employees. These findings emphasize the need to create psychological safety in the workplace to enhance creative output. Through the establishment of a secure atmosphere and a heightened feeling of safety, participative leaders effectively foster a culture of continuous engagement among workers in creative endeavors, therefore reinforcing their creative abilities. Our methodology aligns with previous research that supports the idea that, in the context of the process perspective, leadership has an impact on FRC through individual-level characteristics such as intrinsic motivation and psychological empowerment [22, 41, 50, 63]. The findings of our study contribute to the existing literature that demonstrates the impact of individual and social circumstances on employee creativity, as previously explored by Newman et al. [19] and Wang et al. [41]. Hence, it serves to enhance the acquisition of information, particularly within the realm of social studies and social development. These empirical findings are in line with previous research that finds the evidence for influence FPS has on factors related to followers’ radical creativity [4, 22, 29, 44, 70]. Furthermore, this study enables us to make predictions about human behavior, therefore promoting the establishment of social norms and fostering cooperation. In addition to this, it facilitates the enhancement of the overall well-being of the human population.

Additionally, the results indicate that followers must possess a strong belief in their capabilities and actively cultivate self-determination in order to generate innovative outcomes. One potential rationale for the employee’s function as a mediator in the FPS is rooted in their inherent need to express their creativity, which is supported by their acquired knowledge and abilities. Managers operating within the manufacturing sector must recognize that fostering creativity is a viable strategy for attaining and sustaining a competitive edge. Consequently, these managers need to have a deeper understanding of the potential interplay between PL, FPS, and FRC. Recent research emphasizes the importance of self-efficacy, a major notion in Bandura’s social cognitive theory, in encouraging creativity. High levels of self-efficacy have been associated with better resilience and readiness to participate in novel activities [54]. Furthermore, according to Ryan and Deci’s Self-Determination Theory (SDT), the basic components of self-determination, autonomy, competence, and relatedness, considerably boost intrinsic motivation, a crucial driver of creativity and invention. Followers display better levels of inventive thinking and problem-solving capabilities when they trust in their talents and are driven by internal reasons rather than external rewards [47]. Fostering a sense of self-efficacy and self-determination among followers is therefore critical for obtaining creative results. In accordance with, our study extends and complements these previous findings by empirically demonstrating the key role of participative leadership in inducing employees’ for developing the creative environment and ensuring the psychological safety and enhancing the followers’ radical creativity.

Fourthly, our empirical model examines the collaborative relationship in followers’ manufacturing industries in the relationship between PL, FPS, and FRC. This study addresses the existing research void by extending the previous research and examining the many processes and mechanisms involved in the factors influencing CR and creative behavior. This model has the potential to serve as a starting point for further empirical investigation on CR and contribute significantly to the existing body of literature on CR and FRC within the manufacturing sector.

Fifthly, it is recommended that manufacturing businesses endeavor to educate their leaders and provide incentives for them to cultivate amicable relationships with their subordinates. Effective interpersonal interactions and connections are crucial for fostering strong management practices, which in turn contribute to positive outcomes such as personal growth and the cultivation of innovative abilities among individuals, ultimately leading to enhanced collaboration and engagement within a group. Not only does it facilitate the development of creativity among individuals, but it also enhances the company’s human resources, confidence, and morale, yielding optimal outcomes for the business. Accordingly, this study is unique in moving beyond the prior research on the relationship between participative leadership and creativity, which has been confined to psychological as well as motivational mechanisms to boost employee’s radical creativity [16, 28, 64, 70]and [77].

Sixthly, this research represents a pioneering effort to investigate the relationship between PL and its influence on FRC within the context of manufacturing sectors in Bangladesh. Specifically, it explores the mediating role of FPS and the moderating role of CR in this relationship [76, 78, 79]. The manufacturing sector in Bangladesh is significantly contributing to the country’s economic growth and development. The adoption of a PL style is widely recognized as a crucial factor in fostering followers’ FPS in developed nations. Consequently, it is imperative to extend the application of this leadership approach to developing countries, such as Bangladesh, in order to enhance the motivation and engagement of employees in the manufacturing sector, particularly within the context of the RC environment. Hence, industry pioneers and researchers must recognize the untapped potential of manufacturing sectors in Bangladesh to generate innovative outcomes by answering the research questions. Only through this recognition can a conducive creative atmosphere be established inside firms in developing nations.

Finally, using the SIP theory approach, the researcher discovered that PL positively correlates with FRC through the FPS of followers, which is the first in the published literature to our knowledge that PL is associated with FRC. The findings of this study indicate that participative leaders prioritize their followers’ decision-making abilities and strive to create a safe environment that fosters the development of effective leader-follower interactions [81–83]. It facilitates the generation of innovative and groundbreaking performances by the followers. The present study posits the use of SIP theory as a viable alternative theoretical framework for conducting research on creativity within the context of PL [84, 85]. Consequently, our all objectives has been fulfilled with the support and extending the previous research relating to the theory, context and methodology.

## Conclusions

This study incorporates PL, FPS, and CR to explore social processes that encourage FRC in employees in the manufacturing industry contexts. Regarding the statistical significance of the tested hypothesized model, this research aims to address a specific gap in the existing literature. Additionally, the results of this research may potentially provide a theoretical framework that might effectively communicate a message to the government of Bangladesh and relevant stakeholders, with the aim of propelling Bangladesh towards being a leading nation in the realm of FRC. This research presents novel findings on a hitherto unexplored experience and phenomena within the field of organizational management, economics, and social science development, with a specific focus on the context of FRC in Bangladesh. Motivated by the recognition of the favorable impact of FRC in the manufacturing sector, the industries in Bangladesh must prioritize individual creativity to enhance their innovative capabilities. Hence, the need to foster the creative abilities of followers has significantly grown to preserve originality inside the sector and maintain competitiveness in the marketplace. This research makes a valuable contribution to the development of theoretical frameworks pertaining to creative models within the industrial sector. This model has the potential to serve as a valuable reference for industry executives seeking to strategically modify their organization’s policies and structures to implement a complete management system. The results underscore the practical implications of industry managers’ need to possess knowledge and prioritize the implementation of innovative methods to ensure the long-term viability and endurance of their respective sectors. In general, this research has provided support and developed the understanding and implementation of the concepts of PL, FPS, CR, and FRC to succeed and endure in the current period of competition.

### Theoretical contributions

The dynamics of participatory leadership (PL), psychological safety of followers (FPS), and radical creativity of followers (FRC) are investigated in depth in this research, which reveals significant theoretical implications. Leadership has a significant influence on both FPS and FRC, which establishes it as an essential component in the process of generating a culture inside an organization that is inventive and creative [5, 33, 50, 86]. The engagement of psychological safety as a mediator between PL and FRC is an important finding that highlights the significance of safe and valued working circumstances for the development of creative potential [8, 11, 36, 46, 50].

In line with the social information processing (SIP) theory, the present study asserts that leadership signals have a substantial influence on the feeling of safety and creative participation that workers have in their work. Furthermore, the research casts doubt on the ostensibly positive influence that collaborative relationships (CR) are expected to have on FRC [33, 47, 91]. It presents a more complex picture in which cooperation may sometimes be a hindrance to innovation. This research contributes to the theoretical understanding by illustrating how PL, both directly and indirectly, generates an environment that is psychologically comfortable and creative [36, 46, 87]. Additionally, it provides insights into the complex link that exists between cooperation and creativity.

### Managerial implications

The present study indicates that currently, organizations are using FRC as an effective approach to get a long-lasting competitive edge [22, 33, 57]. Managers should engage in inclusive leadership by actively promoting team engagement and cooperation in decision-making processes. This method aligns with the conclusions of Hoch et al. [20], who demonstrate that participatory leadership plays a crucial role in fostering a creative and psychologically secure environment. Managers should strive to create a conducive climate where workers feel comfortable expressing their opinions and concerns without facing any negative consequences. One may do this by displaying empathy, providing constructive comments, and seeing failures as chances for growth. This approach aligns with the conclusions of Frazier et al. [21] and Wang et al. [35], who both highlight the significance of psychological safety in fostering risk-taking and creativity.

However, managers need to carefully handle team dynamics in order to achieve a mix of joint efforts and individual ideas. This nuanced approach to teamwork is reinforced by Paulus and Nijstad [66], which indicates that while collaboration can be advantageous, it may also hinder innovation under certain conditions. Moreover, recognizing the significance of leadership in creating a psychologically secure atmosphere, it is critical to engage in leadership development programs that focus on these factors. According to Berson et al. [57] training should incorporate practical tools and tactics for creating inclusion and psychological safety. Considering our findings, firms need to frequently examine the effectiveness of their leadership approaches in supporting FPS and FRC. According to Carmeli et al. [23], this can be accomplished through staff surveys, feedback channels, and performance measures. Regular evaluation enables timely modifications and continuing progress in leadership tactics. The manufacturing industry authorities like BGMEA, BKMEA, and other statuary bodies like DCCI and FBCCI need to stimulate an action plan to motivate PL and FRC [68, 88, 89, 100–104].

### Limitations and future directions

While we have made significant contributions, our study does have certain limits. First, the most notable limitation is our study’s cross-sectional design, which prevents us from drawing substantial causal inferences. Therefore, future researchers are encouraged to gather longitudinal data or conduct experimental trials to reproduce our results. The second option is to concentrate only on PL as a prerequisite. In future research, it would be valuable to conduct comparative studies between PL and other leadership styles in order to ascertain any distinctions or supplementary mediation effects. In the third, we just used a single sample frame from Bangladesh. The inclusion of examples from other nations will enhance the recognition of Bangladesh’s followers’ creativity and leadership abilities. We recommend doing this research in different cultural contexts to reinforce or refute our findings. Fourthly, we concentrated on the impact of PL on SIP theory flows and FRC, but we overlooked mechanisms at the team and group levels. Individual and team-level processes may be compared in future empirical research. Future studies should carefully examine how market factors, environmental instability, uncertainty over the environment, and technological upheaval might affect the complex link between PL and FRC displayed by followers. In addition, the negative influence of CR on FRC is a new finding that merits further investigation. It may imply that some sorts of collaborative contexts or how cooperation is carried out may stifle radical creation, a hypothesis that has received little attention in the existing literature.

Furthermore, because most existing research is cross-sectional, longitudinal studies are needed to better understand the long-term benefits of participative leadership on radical creativity. Such research could shed light on how long-term participative leadership techniques impact organizational creativity and innovation.

## Electronic supplementary material

Below is the link to the electronic supplementary material.

Supplementary Material 1

## Data Availability

Data will be provided upon request.
