# Supplementary material for: Participative leadership effects on followers’ radical creativity: the role of psychological safety and collaborative relationship
Source: BMC Psychol. 2025 Jun 4;13:604. doi: 10.1186/s40359-025-02950-3 (PMC12139058; doi:10.1186/s40359-025-02950-3)
Supplement: Supplementary file 1 — Supplementary Material 1 [file 40359_2025_2950_MOESM1_ESM.docx]

**Appendix**

**Appendix-1: Name of Constructs and the Questionnaire**

| Appendix A | | | |
| --- | --- | --- | --- |
| Name of Construct | Items | Description | Reference |
| Participative  Leadership | PL1 | “The supervisor encourages us to express our opinions and suggestions.” | Arnold *et al.*, (2000)  (α= 0.91) |
|  | PL2 | The supervisor listens to my work group's ideas and suggestions |  |
|  | PL3 | The supervisor uses my work group's suggestions to make decisions that affect us |  |
|  | PL4 | The supervisor gives all workgroup members a chance to voice their opinions |  |
|  | PL5 | The supervisor considers my work group's ideas when he/she disagrees with them |  |
|  | PL6 | The supervisor makes decisions that are based only on his/her own ideas |  |
| Followers Psychological  Safety | FPS1 | I am able to bring up problems and tough issues | Edmondson (1999),  (α= 0.77) |
|  | FPS2 | People in this organization do not reject others for being different |  |
|  | FPS3 | It is safe to take a risk in this organization |  |
|  | FPS4 | It is easy for me to ask other members of this organization for help |  |
|  | FPS5 | No one in this organization would deliberately act in a way that undermines my efforts |  |
| Collaborative Relationship | CR1 | There is frequent communication and contact between the two cooperative firms (e.g., visits to each other’s firms, meetings, written and telephone communications) | Anderson and Weitz (Anderson and Weitz, 1992) and Heide and John (1992)  (α =.840) |
|  | CR2 | Both our buyers and we are generally honest and truthful with each other. |  |
|  | CR3 | The number of years since the suppliers cooperating with the buyers. |  |
| Follower Radical Creativity | FRC1 | “Is a good source of highly creative ideas" | Madjar *et al* (Madjar *et al.*, 2011) and Baer (2012)  (α =0.87) |
|  | FRC2 | “Demonstrates originality in his/her work,” |  |
|  | FRC3 | “Suggests radically new ways for doing advertising.” |  |
|  | FRC4 | “developed ideas that imply substantial departures from existing product and service lines” |  |
|  | FRC5 | “developed ideas that make existing knowledge about current products/services obsolete”; |  |
|  | FRC6 | “developed breakthrough ideas—not minor changes to existing products/services.” |  |
